# Psychometric Properties of the Italian Version of the Young Schema Questionnaire L-3: Preliminary Results

**DOI:** 10.3389/fpsyg.2018.00312

**Published:** 2018-03-27

**Authors:** Aristide Saggino, Michela Balsamo, Leonardo Carlucci, Veronica Cavalletti, Maria R. Sergi, Giorgio da Fermo, Davide Dèttore, Nicola Marsigli, Irene Petruccelli, Susanna Pizzo, Marco Tommasi

**Affiliations:** ^1^School of Medicine and Health Sciences, Università degli Studi ‘G. d'Annunzio’ Chieti - Pescara, Chieti, Italy; ^2^Center for the Study of Personality, Napoli, Italy; ^3^IPSICO - Istituto di Psicologia e Psicoterapia Comportamentale e Cognitiva, Firenze, Italy; ^4^Azienda USL di Pescara, Pescara, Italy; ^5^Centro di Psicologia Clinica, Pescara, Italy; ^6^Department of Health Sciences, Florence University, Florence, Italy; ^7^Department of Human Sciences and Society, Enna “Kore” University, Enna, Italy; ^8^Cognitive and Behavioral Therapy Institute, Padua, Italy

**Keywords:** Young Schema Questionnaire L3, reliability, validity, schema therapy, factor analysis, statistical

## Abstract

Schema Therapy (ST) is a well-known approach for the treatment of personality disorders. This therapy integrates different theories and techniques into an original and systematic treatment model. The Young Schema Questionnaire L-3 (YSQ-L3) is a self-report instrument, based on the ST model, designed to assess 18 Early Maladaptive Schemas (EMSs). During the last decade, it has been translated and validated in different countries and languages. This study aims to establish the psychometric properties of the Italian Version of the YSQ-L3. We enrolled two groups: a clinical (*n* = 148) and a non-clinical one (*n* = 918). We investigated the factor structure, reliability and convergent validity with anxiety and depression between clinical and non-clinical groups. The results highlighted a few relevant findings. Cronbach's alpha showed significant values for all the schemas. All of the factor models do not seem highly adequate, even if the hierarchical model has proven to be the most significant one. Furthermore, the questionnaire confirms the ability to discriminate between clinical and non-clinical groups and could represent a useful tool in the clinical practice. Limitations and future directions are discussed.

## Introduction

Schema Therapy (ST; Young, 1994; Young et al., 2003) provided an innovative approach to psychotherapy aiming to treat patients with chronic psychological problems. Several studies showed that ST is an evidence-based treatment for personality disorders (e.g., Giesen-Bloo et al., 2006; Gude and Hoffart, 2008; Farrell et al., 2009; Nadort et al., 2009; Sempertegui et al., 2013; Bamelis et al., 2014), as well as for anxiety and depressive disorders (Balsamo, 2010, 2013; Renner et al., 2013; Malogiannis et al., 2014; Balsamo et al., 2015c; for a review, Hawke et al., 2011) and eating disorders (Waller et al., 2007). ST is currently being implemented within the mental health services of several nations, such as Denmark (Bach et al., 2015).

ST was developed as the clinical implication of Young (1994) schema theory. It is an integrative therapy, mixing elements of different approaches such as Cognitive-Behavioral Therapy, Gestalt therapy, Attachment Theory, Object Relations Theory and emotional-focused models (Young, 1994). Influenced by these theories, Young and colleagues (Young, 1994; Young et al., 2003) developed the “Early Maladaptive Schemas” (EMSs) concept, as a broad, pervasive, trait-like, cognitive and emotional self-defeating pattern, concerning beliefs about the self, others and the future. According to the ST model, EMSs derive from early childhood noxious experiences with primary caregivers and are established by unmet core emotional needs (Young et al., 2003), as well as from peer relations during childhood and adolescence (Mash and Dozois, 2003; Renner et al., 2013). Little evidence seemed to support the association between early relational experiences and EMSs (e.g., Muris, 2006; Wright, 2007) as well as between schemas and psychopathology symptoms such as depression and anxiety in adulthood (Halvorsen et al., 2009; Hawke et al., 2011; Renner et al., 2012; Riso et al., 2017), or in youth (Van Vlierberghe et al., 2010; Balsamo et al., 2015c), even though some authors maintained that infant attachment may be an overrated predictor (e.g., Meins, 2017).

The current list of EMSs consists of 18 schemas, which have been identified in the general populations, as well as in clinical groups (Young, 1994). The 18 EMSs have been grouped into five broad categories of unmet emotional needs called “schema domains.” These broad categories are: disconnection and rejection, impaired autonomy and performance, other directedness, over-vigilance and inhibition and impaired limits (Young et al., 2003).

The *Young Schema Questionnaire* (YSQ; Young and Brown, 1994) is a self-report measure developed to assess EMSs within the ST. It is used as a clinical instrument in psychotherapy and as a research measure in developmental psychopathology studies. The first YSQ-Long Form consisted of 205 items, representing the 16 EMSs listed by the authors. After a psychometric revision of the EMSs (Schmidt et al., 1995), Young et al. (2003) 18 EMS were operationally defined and a new YSQ-Long Form was developed. This Third Edition (YSQ-L3; Young and Brown, 1994), consisted of 232 items. According to a literature review (Oei and Baranoff, 2007), although the Third Edition underwent many revisions, no consistent factor structures emerged for the YSQ-L3.

Whereas the psychometric properties of the YSQ were tested in different languages and groups (clinical and non-clinical participants), almost all of the studies employed the short form or the previous forms, which are not comparable with the YSQ L3 form. Furthermore, to the best of our knowledge, this is the first study in Italy that explores the YSQ-L3 structural validity by means of Confirmatory Factor Analysis.

In this study, we examined the reliability and structural validity of the 18 schema scales, as measured by the YSQ-L3. We specifically tested its structural validity by investigating whether the five correlated first-order factor structure, proposed by the test developers (Young et al., 2003), could be replicated in two Italian groups (clinical and non-clinical subjects) by Confirmatory Factor Analysis, as well as the one-factor model, recently found in the Italian version of the YSQ-L3 via Exploratory Factor Analysis (see Saggino et al., 2017). Since the findings resulting from current literature on the YSQ-L3'slatent factor structure were inconclusive (Oei and Baranoff, 2007), we also tested a bi-factor model, strongly suggested by Kriston et al. (2012) for the YSQ-SF3, in which all the 18 schemas loaded each on own domain and on one global factor, called “Psychopathology.”

Finally, we tested the second-order model with five first-order factors according to Young's model as well as a general second-order factor.

We also investigated the reliability of the YSQ-L3, as well as its convergent validity by computing associations between the YSQ-L3 and concurrent measures of anxiety and depression. In addition, we carried out a Multigroup Confirmatory Factor Analysis (MG-CFA) to test measurement invariance of the YSQ-L3 with respect to groups of subjects with and without psychological syndromes. Furthermore, false positive (FP) risk values were calculated to discriminate between non-clinical and clinical subjects.

## Materials and methods

### Participants

Participants ranged between the ages of 18 and 89 and had the capacity to complete self-administered questionnaires. This group was the same used for the Italian norms in a previous study (Saggino et al., 2017). Inclusion criteria for the clinical group were: existence of a psychiatric diagnosis and age = or > 17 years old. Exclusion criteria included ongoing psychotic symptoms, serious physical illnesses and central nervous system major disorders (e.g., Alzheimer's disease and Parkinson's disease). Participants were 1,112 Italian subjects: 157 clinical and 955 community participants. Forty-six were excluded from the analyses: 9 clinical and 37 non-clinical subjects were removed because they had missing values ≥10% at EMSs. Missing values rated below 10%, were replaced with the average values of each schema.

The *clinical group* was formed by 148 outpatients of which 52 females (35.1%) and 96 males (64.9%). The group's mean age was 37.92 (*SD* = 10.43; range = 18–64 years). The mean age for men was 38.28 years (*SD* = 9.96), and 37.25 years for women (*SD* = 11.80). No significant age by gender interaction effect was found [*F*_(1, 146)_ = 0.328, *p* = 0.568]. The mean years of education was 12.47 (*SD* = 3.23; range = 8–20 years): 11.89 (*SD* = 3.08) for males and 13.60 years (*SD* = 3.22) for females. A significant years of education by gender interaction effect was found [*F*_(1, 136)_ = 9.17, *p* = 0.003].

The *non-clinical* group was formed by 918 subjects of which 522 females (56.9%) and 396 males (43.1%). The group's mean age was 29.85 years (*SD* = 12.56; range = 18–89 years): 31.09 years (*SD* = 13.09) for males, and 28.92 years (*SD* = 12.35) for females. There was a statistically significant difference in age between males and females [*F*_(1, 912)_ = 6.58, *p* = 0.010]. The mean years of education was 13.63 years (*SD* = 3.36; range 5–25 years): 13.45 years (*SD* = 3.34) for males and 13.77 years (*SD* = 3.38) for females. No statistically significant difference was found in years of education between males and females [*F*_(1, 892)_ = 1.89, *p* = 0.169]. All subjects were white.

The clinical group was recruited through private practice (*N* = 49; 33.1%), private psychiatric hospitals (*N* = 13; 8.8%), public psychiatric hospital (*N* = 23; 15.5%) and mental health departments (*N* = 63; 42.6%). Diagnoses were conducted according to the Diagnostic and Statistical Manual of Mental Disorders standards (DSM-IV-TR; American Psychiatric Association, 2000) by accredited psychiatrists and psychologists. The patients included in this group were diagnosed as follows: 56.8% (*N* = 84) received a diagnosis of a disorder on DSM-IV-TR Axis I, 15.5% (*N* = 23) received a diagnosis of a disorder on DSM-IV-TR Axis II and 20.9% (*N* = 31) received a comorbid diagnosis Axis I/Axis II. For 6.8% (*N* = 10) of the clinical group there was no information available about the diagnosis.

The non-clinical group was recruited through advertisements posted in established community groups (e.g., youth centers, church groups, university student associations). Study participants contributed voluntarily and anonymously. Each participant anonymously completed the questionnaire packet and gave informed consent prior to being included in the study.

131 non-clinical participants (94 females and 37 males, mean age = 22.15 and *SD* = 4.37) filled out the YSQ-L3 again after 1 month (T0); 72 non-clinical participants (57 females and 15 males, mean age = 20.86 *SD* = 2.97) filled out the YSQ-L3 again 1 month after the first retest (T1); 40 non-clinical participants (28 females and 12 males, mean age = 21.75 *SD* = 3.71) filled out the YSQ-L3 1 month after the second retest (T2).

### Instruments

All participants were administered the Italian versions of the Young Schema Questionnaire Long Form, Third Edition (YSQ-L3), the Teate Depression Inventory (TDI), the State-Trait Inventory for Cognitive and Somatic Anxiety Trait Scale (STICSA). All respondents completed paper-and-pencil versions of the questionnaires in a fixed order (a socio-demographic checklist, the YSQ L3, the TDI, and the STICSA) on site at established community groups. The protocol was administered by licensed psychologists who received a brief training wherein the objectives of the research, characteristics of the instruments administered and information about common issues in the psychological assessment of adults were explained. Informed consent was obtained from every single participant included in the study, in accordance with the Ethical Standards of the Helsinki Declaration.

#### Young schema questionnaire-long form, third edition

The YSQ-L3 (Young et al., 2003) is a 232-item self-report tool developed to assess 18 EMSs. The Italian version of the questionnaire is in the Appendix of the Young et al. (2003)'s Italian book. Participants are asked to rate each statement on a 6-point Likert scale ranging from 1 (“it is completely untrue for me”) to 6 (“it describes me perfectly”). Items are clustered by 18 scales and grouped into five domains, bringing together the EMSs that tend to develop together: Disconnection/Rejection (Abandonment, Mistrust/ Abuse, Emotional Deprivation, Defectiveness/Shame, Social Isolation/Alienation); Impaired Autonomy/Performance (Dependence/Incompetence, Vulnerability to Harm or Illness, Enmeshment/Undeveloped Self, Failure); Impaired Limits (Entitlement/Grandiosity, Insufficient Self-Control/Self-Discipline); Other-Directedness (Subjugation, Self-Sacrifice, Approval-Seeking/Recognition-Seeking); and Overvigilance/Inhibition (Negativity/Pessimism, Emotional Inhibition, Unrelenting Standards/Hypercriticalness, Punitiveness). A sum or a mean score is calculated for each EMS, a higher score representing a higher endorsement of the EMS in question. YSQ has demonstrated adequate test–retest reliability and internal consistency, as well as convergent and discriminant validity (Young et al., 2003). Results attained from several YSQ studies support its validity as an EMS measure (Lee et al., 1999; Stopa et al., 2001; Hoffart et al., 2005). Cronbach's α coefficients for this current study are reported in **Table 2**. All the statistical analyses in this research were based on the mean score of each EMS.

#### State-trait inventory for cognitive and somatic anxiety

The STICSA (Ree et al., 2008; Italian version see Balsamo et al., 2015a, 2016) is a 21-item measure designed to assess cognitive and somatic symptoms, both on Trait and State variations. In the trait anxiety subscale, the subject rates how often a statement is true *in general* (on a four-point Likert-type scale from “1-almost never at all” to “4-almost always”), whereas she/he rates how she/he feels *at the moment of assessment* (on a four-point Likert-type scale from “1-not at all” to “4-very much”) in the state anxiety subscale. In total, the overall scale is made up of four subscales: State–Somatic (SS), Trait–Somatic (TS), State–Cognitive (SC), and Trait–Cognitive (TC).

The STICSA was developed to address the psychometric limitations of existing anxiety measures, especially as far as their extensive overlapping depression (Caci et al., 2003; Balsamo et al., 2013a; Roberts et al., 2016). The factor structure showed strong support and the total scale and subscales exhibited high internal consistencies, as well as construct consistent correlations in patients, controls, and community groups (Grös et al., 2007; Ree et al., 2008; Van Dam et al., 2013; Saggino et al., 2017). Cronbach's α coefficients for this current study are from 0.812 (State-Somatic) to 0.926 (State).

#### Teate depression inventory

The TDI (Balsamo and Saggino, 2013, 2014; Balsamo et al., 2014) is a 21-itemself-report instrument designed to assess Major Depressive Disorder as specified by the latest edition of the DSM (American Psychiatric Association, 2013). It was developed via Rasch logistic analysis of responses (Rasch, 1960), within the framework of Item Response Theory, in order to overcome inherent psychometric weaknesses of existing depression measures, including the BDI-II (Balsamo and Saggino, 2007). Each item is rated on a 5-point Likert-type scale, ranging from 0 (always) to 4 (never). Growing literature suggests that the TDI has strong psychometric properties in both clinical and non-clinical groups, including an excellent Person Separation Index, no evidence of bias due to item-trait interaction, good discriminant and convergent validity and control of major response sets (Balsamo et al., 2013b, 2015a,b,c; Innamorati et al., 2013). In a recent study, three cut-off scores were recommended in terms of sensitivity, specificity and classification accuracy to screen for varying levels (minimal, mild, moderate and severe) of depression severity in a group of patients diagnosed with Major Depressive Disorder (Balsamo and Saggino, 2014). In our groups, Cronbach's alpha was 0.943 for the clinical participants and 0.917 for the non-clinical group.

### Data analysis

#### Descriptive statistics

The 18 EMSs were preliminarily submitted to analyses in order to check the normal distribution by computing means, standard deviations and indices of skewness and kurtosis. Inspection of skewness and kurtosis indices indicated that departures from normality were not severe according to West et al. (1995) with only a few exceptions. Thus, no variable transformations were deemed necessary. Statistical analyses were performed with IBM SPSS.

#### Reliability, and convergent validity analysis of the YSQ-L3

In order to investigate the psychometric properties of the YSQ-L3, we assessed internal consistency of its scales using Cronbach's alphas indices separately for the two groups. The two-way mixed effects ICC (Intraclass-Correlation; Shrout and Fleiss, 1979; McGraw and Wong, 1996) was used to assess the 3-month test–retest stability (T0, T1, T2) of each EMS' schema on a group formed by 40 non-clinical subjects. The strong reduction of subjects is due to mortality or to the fact that many subjects refused to repeat test administration. Since the Shrout and Fleiss' (1979) ICC rules of thumb were criticized (Hopkins, 2000), we considered the following values as a general rule: ≥ 0.90 high, between 0.80 and 0.90 moderate, and ≤0.80 insufficient (Vincent, 1999).

The convergent validity of the YSQ-L3 schemas was investigated by computing Pearson's *r* correlation coefficients with well-established depression and anxiety measures (TDI and STICSA, respectively). Error α was adjusted with Bonferroni's correction. These statistical analyses were performed with IBM SPSS.

#### Confirmatory factor analyses of the YSQ-L3

Different Confirmatory Factor Analyses (CFAs) were performed separately for the clinical and non-clinical participants. Due to a slight deviation from multivariate normality all analyses were carried out using robust maximum-likelihood estimation methods. Given the heterogeneity of the results reported in literature regarding the latent factor structure of Young's EMSs (for a review, see Kriston et al., 2012), most of which referred to the different YSQ versions, we compared five alternative factor models for the Italian version of the YSQ-L3. These versions were: (1) the one-factor model (1F model), in which all 18 schemas were forced to load on a single higher order factor (Saggino et al., 2017); (2) the five correlated first-order factors model (5F-correlated model), based on Young's original theoretical model (Young et al., 2003); (3) the five not correlated first-order factors model, according to Young's model, without correlations between factors (5F-not correlated model); (4) the bi-factor model (bi-factor model), strongly suggested by Kriston et al. (2012), in which all of the 18 EMS schemas loaded each on own domain and on one global factor, called “Psychopathology”; (5) finally, the second-order model, with the five first-order factors model, according to Young's model, and a general second-order factor.

The goodness-of-fit indices to test model validity were the Satorra-Bentler χ^2^, the ratio χ^2^/df, the Comparative Fit Index (CFI), the Tucker-Lewis fit index (TLI), the Root Mean Square Error of Approximation (RMSEA) and the corresponding confidence interval (90% RMSEA). Models with an acceptable fit should have χ^2^/df < 3, RMSEA <0.08, and CFI and TLI >0.95 (Hu and Bentler, 1999; Schermelleh-Engel et al., 2003).

#### Measurement invariance of the YSQ-L3 between non-clinical and clinical groups

We performed a Multigroup Confirmatory Factor Analysis (MG-CFA) to test measurement invariance of the YSQ-L3 with respect to groups of subjects with and without psychological syndromes on a set of nested models (Meredith, 1993; Saggino et al., 2017):

1. The baseline configural invariance model (M1) in which the same factorial pattern was specified for each group, but with loadings and intercepts free to vary across groups;
2. The metric invariance model (M2), wherein loadings were constrained to be equal across groups;
3. The scalar invariance model (M3), wherein factor loadings and intercepts were constrained to be equal across groups;

There is also the model for testing strict invariance (loadings, intercepts and residual variances were constrained to be equal across groups), but strict invariance is not fundamental for the validity of the model. Model fit was assessed using the χ^2^ statistical test, the χ^2^/df, the RMSEA, the 90% CI of RMSEA, the SRMR, the TLI and the CFI.

Difference between CFIs (ΔCFI) of nested models was estimated for testing measurement invariance. A value of ΔCFI smaller than or equal to |0.01| (in absolute values) indicates that the null hypothesis of invariance should not be rejected (Cheung and Rensvold, 2002). Tests which have scalar invariance are considered consistent tests, because unaffected by group characteristics (Meredith, 1993). If multigroup invariance is confirmed with models M2 or M3, we also tested if factor means are different across groups by setting a model wherein the factor means are zero in all groups (M4). We estimated the difference between the chi-square value of M4 and that of model M2 or M3. If the value of the difference is not significant, factor means can be considered equal across groups. CFAs and MG-CFA were performed using M-Plus 7.0 (Muthén and Muthén, 2012).

Furthermore, false positive (FP) risk values were calculated for each YSQ-L3 schema and domain. FP risks are determined by the False Positive Rate (FPR), which is the ratio between the probability of False Positives (FPs) and the sum of FPs and True Positives (TPs). Because a clinical test such as the YSQ-L3 has to discriminate between non-clinical and clinical subjects, we must estimate FPR ratio, instead of using the criterion of rejecting the null hypothesis with a first-type error probability value of 0.05, in order to attain the correct percentage of risk to make FPs using test scores (Colquhoun, 2014). All of the analyses were based on the standardized scores for any schema and on the factor scores, for any latent domain.

All missing data were substituted by the serial mean. The work of Chen et al. (2012) showed that with a percentage of missing data below 20% there is no reduction of fit indices. The model fit decreases as the number of missing data gets larger. The authors suggest that when the percentage of missing data is higher than 30%, both the serial mean and the trend missing imputation methods offer a better model fit than the other available methods. Because the missingness in our data was always below 10%, we therefore used the Serial Mean method.

## Results

### Descriptive statistics of the YSQ-L3

Descriptive statistics of the 18 EMS, the TDI and the STICSA State-Trait; somatic and cognitive scales in the Italian clinical and non-clinical groups are displayed in Table 1.

**Table 1:** Descriptive Statistics of the EMS, TDI, and STICSA for non-clinical (*n* = 918) and clinical sample (*n* = 148).

**EMS**	**Non-clinical (*N* = 918)**				**Clinical (*N* = 148)**			
	Mean^*^	SD^*^	**Skewness**	**Kurtosis**	Mean^*^	SD^*^	**Skewness**	**Kurtosis**
Emotional deprivation	2.10	0.92	1.133	1.018	2.78	1.16	0.583	−0.240
Abandonment	2.37	0.84	0.741	0.337	2.87	1.07	0.507	−0.279
Mistrust/Abused	2.28	0.75	0.787	0.653	2.59	0.94	0.472	−0.212
Social isolation	1.92	0.85	1.346	1.976	2.43	1.12	0.608	−0.532
Defectiveness	1.68	0.67	2.030	6.202	2.11	0.92	1.175	1.185
Failure to achieve	1.84	0.84	1.660	3.573	2.21	1.07	0.981	0.355
Dependence	1.80	0.70	1.315	1.933	2.31	1.03	1.052	1.052
Vulnerability	2.05	0.80	1.267	1.937	2.43	1.04	1.119	1.210
Enmeshment	2.00	0.69	0.993	1.395	2.36	0.96	0.834	0.237
Subjugation	2.13	0.79	1.056	1.421	2.56	0.96	0.684	0.521
Self–sacrifice	3.08	0.87	0.343	−0.207	3.05	0.90	0.310	−0.109
Approval–seeking	2.18	0.84	1.019	1.550	2.51	0.97	0.528	−0.202
Insufficient self–control	2.20	0.73	0.685	0.433	2.59	0.96	0.469	−0.331
Entitlement	2.55	0.78	0.489	0.305	2.69	0.89	0.218	−0.574
Unrelenting standards	2.77	0.82	0.621	0.319	2.76	0.82	0.171	−0.303
Emotional inhibition	2.24	0.91	1.025	1.127	2.64	1.09	0.516	−0.217
Negativism	2.34	0.94	0.786	0.347	2.76	1.05	0.225	−0.629
Self-punitiveness	2.55	0.80	0.483	0.323	2.63	0.85	0.078	−0.427
TDI	28.15	13.17	0.356	−0.214	35.10	16.87	0.164	−0.507
STICSA-trait	35.62	10.18	0.718	−0.044	39.53	12.83	0.839	0.486
STICSA-trait, somatic	17.36	5.20	0.939	0.507	18.32	6.92	1.340	1.540
STICSA–trait, cognitive	18.25	5.99	0.651	−0.211	21.21	7.15	0.313	−0.801
STICSA-state	32.04	10.20	0.692	1.279	35.43	13.14	1.012	2.123
STICSA-state, somatic	15.51	4.81	1.534	2.574	16.33	6.76	1.935	4.399
STICSA-state, cognitive	16.50	5.99	1.047	0.462	18.99	7.30	0.469	−0.048

* *Means and Standard Deviations are based on means of EMS*.

As shown in Table 1, in our sample all the EMS schemas exhibited no absolute value of skewness larger than 2, neither absolute values of kurtosis larger than 7, in both groups, excepting for Defectiveness which presented a skewness corresponding to 2.030 in the non-clinical group, according to the guidelines recommended by West et al. (1995). A similar trend of normality distribution was observed for the TDI and the STICSA scales and subscales.

### Reliability, and convergent validity analysis of the YSQ-L3

As shown in Table 2, internal consistency reliability of the 18 EMS was high (range α_clinical_ = 0.804–0.921 and α_non−clinical_ = 0.834–0.941).

**Table 2:** Cronbach alpha and test-retest Reliability of the 18 EMS of the YSQ-L3.

	**T0**		**T1^†^**		**T2^†^**		**ICC**	**ICC 95% CI**		**F**	α	
	***M***	***SD***	***M***	***DS***	***M***	***DS***		**Lower**	**Upper**		**Clinical**	**Non-clinical**
Emotional deprivation	2.13	0.91	2.09	1.01	2.30	1.09	0.925	0.878	0.957	38.148^*^	0.896	0.895
Abandonment	2.53	0.83	2.35	0.84	2.37	0.85	0.783	0.666	0.869	11.799^*^	0.894	0.918
Mistrust/Abused	2.37	0.73	2.17	0.80	2.28	0.80	0.786	0.671	0.872	12.042^*^	0.893	0.911
Social isolation	1.99	0.85	1.96	0.94	2.10	1.06	0.869	0.792	0.924	20.947^*^	0.883	0.899
Defectiveness	1.73	0.65	1.70	0.71	1.85	0.80	0.889	0.822	0.936	25.087^*^	0.905	0.911
Failure to achieve	1.90	0.86	1.89	0.80	1.84	0.79	0.703	0.558	0.817	8.103^*^	0.918	0.901
Dependence	1.86	0.64	1.71	0.65	1.70	0.63	0.768	0.646	0.860	10.930^*^	0.921	0.941
Vulnerability	2.12	0.80	1.98	0.87	2.05	0.83	0.856	0.770	0.916	18.785^*^	0.872	0.907
Enmeshment	2.08	0.70	1.87	0.68	1.91	0.72	0.781	0.663	0.868	11.676^*^	0.804	0.875
Subjugation	2.19	0.77	2.05	0.76	2.15	0.92	0.715	0.574	0.825	8.511^*^	0.841	0.858
Self-sacrifice	3.19	0.85	2.94	0.91	2.96	0.89	0.854	0.769	0.914	18.596^*^	0.906	0.895
Approval-seeking	2.20	0.78	2.14	0.81	2.23	0.88	0.766	0.643	0.859	10.811^*^	0.916	0.914
Insufficient self-control	2.25	0.69	2.11	0.71	2.15	0.63	0.791	0.678	0.875	12.344^*^	0.862	0.894
Entitlement	2.61	0.73	2.39	0.76	2.37	0.76	0.766	0.634	0.859	10.810^*^	0.823	0.834
Unrelenting standards	2.82	0.82	2.60	0.82	2.58	0.78	0.802	0.694	0.882	13.185^*^	0.886	0.868
Emotional inhibition	2.37	0.94	2.17	0.95	2.40	0.95	0.785	0.670	0.871	11.970^*^	0.840	0.876
Negativism	2.45	0.92	2.24	0.97	2.24	0.90	0.780	0.663	0.868	11.663^*^	0.903	0.893
Self-punitiveness	2.65	0.78	2.50	0.83	2.54	0.78	0.776	0.657	0.865	11.390^*^	0.881	0.874

* p < 0.01. N = 40; ICC, Intraclass Correlation Coefficient; CI, Confidence Interval.

† *Rating at 1-month distance*.

As shown in Table 2, the ICC estimates were similar in value, for each Young's schema. The Emotional Deprivation schema ICC was 0.925, with 95% confidence interval from 0.878 to 0.957 [*F*_(39, 78)_ = 38.148, *p* < 0.001]. A moderate reliability degree was also found for Social Isolation [ICC = 0.869; 95%, CI = 0.792–924; *F*_(39, 78)_ = 20.947, *p* < 0.001], Defectiveness [ICC = 0.889; 95%, CI = 0.822–936; *F*_(39, 78)_ = 25.087, *p* < 0.001], Vulnerability [ICC = 0.856; 95% CI = 0.770–916; *F*_(39, 78)_ = 18.785, *p* < 0.001], Self-Sacrifice [ICC = 0.854; 95%, CI = 0.769–914; *F*_(39, 78)_ = 18.596, *p* < 0.001], and Unrelenting Standards [ICC = 0.802; 95%CI = 0.694–882; *F*_(39, 78)_ = 13.185, *p* < 0.001]. The remaining EMS schemas showed ICC values considered as insufficient (cut-off ≤ 0.80; Vincent, 1999), ranging from 0.703 (Failure to Achieve) to 0.791 (Insufficient Self-control).

Table 3 shows the correlations among the 18 EMS, measures of depression (TDI) and trait and state anxiety (STICSA, with its subscales). As expected, all of the EMS in general showed an average to high correlation with the TDI and the STICSA scales both in the clinical and in the non-clinical groups.

**Table 3:** correlations between the 18 schemas of the YSQ-L3 with TDI and STICSA for non-clinical and clinical sample.

	**TDI**		**STICSA-trait**		**STICSA-trait, somatic**		**STICSA-trait, cognitive**		**STICSA-state**		**STICSA-state, somatic**		**STICSA-state, cognitive**	
	**Non-clinical**	**Clinical**	**Non-clinical**	**Clinical**	**Non-clinical**	**Clinical**	**Non-clinical**	**Clinical**	**Non-clinical**	**Clinical**	**Non-clinical**	**Clinical**	**Non-clinical**	**Clinical**
Emotional deprivation	0.347^**^	0.283	0.365^**^	0.269	0.256^**^	0.238	0.399^**^	0.250	0.362^**^	0.318	0.227^**^	0.289	0.384^**^	0.261
Abandonment	0.478^**^	0.535^**^	0.571^**^	0.526^**^	0.442^**^	0.464^**^	0.588^**^	0.495^**^	0.511^**^	0.443^**^	0.362^**^	0.375	0.533^**^	0.407^**^
Mistrust/Abused	0.335^**^	0.415^**^	0.436^**^	0.437^**^	0.325^**^	0.400	0.458^**^	0.397	0.372^**^	0.375	0.258^**^	0.311	0.404^**^	0.335
Social isolation	0.449^**^	0.561^**^	0.465^**^	0.466^**^	0.307^**^	0.380	0.523^**^	0.468^**^	0.392^**^	0.417^**^	0.268^**^	0.344	0.440^**^	0.389
Defectiveness	0.472^**^	0.546^**^	0.504^**^	0.533^**^	0.376^**^	0.443^**^	0.530^**^	0.528^**^	0.461^**^	0.509^**^	0.365^**^	0.420^**^	0.477^**^	0.496^**^
Failure to achieve	0.509^**^	0.585^**^	0.482^**^	0.513^**^	0.333^**^	0.415^**^	0.530^**^	0.518^**^	0.438^**^	0.486^**^	0.306^**^	0.422^**^	0.482^**^	0.444^**^
Dependence	0.499^**^	0.633^**^	0.542^**^	0.577^**^	0.414^**^	0.491^**^	0.563^**^	0.559^**^	0.494^**^	0.531^**^	0.390^**^	0.432^**^	0.510^**^	0.508^**^
Vulnerability	0.411^**^	0.468^**^	0.586^**^	0.617^**^	0.471^**^	0.576^**^	0.586^**^	0.548^**^	0.505^**^	0.473^**^	0.393^**^	0.442^**^	0.527^**^	0.384
Enmeshment	0.263^**^	0.411^**^	0.384^**^	0.501^**^	0.328^**^	0.448^**^	0.368^**^	0.464^**^	0.350^**^	0.478^**^	0.301^**^	0.370	0.338^**^	0.458^**^
Subjugation	0.429^**^	0.513^**^	0.496^**^	0.581^**^	0.355^**^	0.526^**^	0.534^**^	0.532^**^	0.461^**^	0.561^**^	0.338^**^	0.484^**^	0.476^**^	0.494^**^
Self-sacrifice	0.191^**^	0.221	0.354^**^	0.431^**^	0.278^**^	0.411^**^	0.360^**^	0.374	0.331^**^	0.428^**^	0.241^**^	0.396	0.358^**^	0.333
Approval-seeking	0.350^**^	0.467^**^	0.413^**^	0.614^**^	0.278^**^	0.564^**^	0.460^**^	0.555^**^	0.365^**^	0.550^**^	0.240^**^	0.494^**^	0.401^**^	0.465^**^
Insufficient self-control	0.484^**^	0.573^**^	0.520^**^	0.527^**^	0.370^**^	0.400	0.562^**^	0.558^**^	0.462^**^	0.462^**^	0.318^**^	0.318^**^	0.490^**^	0.479^**^
Entitlement	0.261^**^	0.444^**^	0.342^**^	0.442^**^	0.241^**^	0.389	0.372^**^	0.415^**^	0.279^**^	0.370	0.187^**^	0.293	0.294^**^	0.334
Unrelenting standards	0.125	0.207	0.283^**^	0.414^**^	0.212^**^	0.394	0.297^**^	0.362	0.231^**^	0.417^**^	0.157	0.430^**^	0.252^**^	0.290
Emotional inhibition	0.407^**^	0.544^**^	0.537^**^	0.597^**^	0.416^**^	0.512^**^	0.552^**^	0.575^**^	0.454^**^	0.526^**^	0.339^**^	0.467^**^	0.479^**^	0.442^**^
Negativism	0.525^**^	0.627^**^	0.639^**^	0.683^**^	0.468^**^	0.562^**^	0.680^**^	0.681^**^	0.554^**^	0.566^**^	0.378^**^	0.470^**^	0.605^**^	0.511^**^
Self-punitiveness	0.310^**^	0.446^**^	0.421^**^	0.572^**^	0.303^**^	0.462^**^	0.452^**^	0.580^**^	0.384^**^	0.537^**^	0.288^**^	0.464^**^	0.399^**^	0.463^**^

** *p < 0.01. Error α was adjusted with Bonferroni's correction. TDI, Teate Depression Inventory; STICSA, State-Trait Inventory for Cognitive and Somatic Anxiety*.

### Confirmatory factor analyses of the YSQ-L3

Table 4 shows the goodness-of-fit indexes of the five structural models tested both for the non-clinical and the clinical groups. Although the bi-factor model has the best fit, as far as both the non-clinical and the clinical group, it exhibits many flaws at a more detailed level.

**Table 4:** Goodness-of-fit indexes of the five models tested in the CFAs both for the non-clinical (*n* = 918) and the clinical sample (*n* = 148).

**CFA models**	**χ^2^**	**df**	**χ^2^/df**	**CFI**	**TLI**	**RMSEA**	**90% RMSEA**	**SRMR**	**AIC**	**BIC**
**NON-CLINICAL**											
1F model	1794.657	135	13.294	0.785	0.756	0.116	0.111–0.121	0.071	31151.93	31412.33
5F-correlated model	1499.681	125	11.997	0.822	0.782	0.109	0.105–0.114	0.066	30706.75	31015.37
5F-not correlated model	3816.683	136	28.064	0.523	0.463	0.172	0.167–0.176	0.389	33932.87	34188.45
Bi-factor model	935.738	107	8.745	0.893	0.846	0.092	0.086–0.097	0.044	29862.44	30257.86
Second-order model	1527.210	130	11.748	0.819	0.787	0.108	0.103–0.113	0.068	30758.38	31042.89
**CLINICAL**											
1F model	401.318	135	2.973	0.841	0.820	0.115	0.103–0.129	0.060	5709.360	5871.209
5F-correlated model	296.916	125	2.375	0.897	0.874	0.096	0.082–0.111	0.051	5591.381	5783.202
5F-not correlated model	815.722	136	5.998	0.594	0.543	0.184	0.172–0.196	0.457	6218.797	6377.649
Bi-factor model	244.283	107	2.283	0.918	0.883	0.093	0.078–0.109	0.037	5526.267	5772.039
Second-order model	308.082	130	2.370	0.894	0.875	0.096	0.082–0.110	0.053	5598.831	5775.666

The loadings of the Disconnection/Rejection domain are especially not significant for the Abandonment and the Defectiveness/Shame schema in the clinical group; the loadings of the Impaired Autonomy/Performance domain are not significant for all of the four schemas in the clinical group and are not significant for the Failure schema in the non-clinical group; the loadings of the Other-Directedness domain are not significant for the Subjugation and for the Approval-Seeking/Recognition-Seeking schema in the clinical group; the loading of the Impaired Limits domain on the Insufficient Self-Control/Self-Discipline schema is not significant in the clinical group; the loadings of the Overvigilance/Inhibition domain on the Emotional Inhibition, and the Unrelenting Standards/Hypercriticalness schema are not significant in the clinical participants. Not-significant loadings mean that the bi-factor model does not provide adequate measurement properties. Table 5 shows the loadings of each schema in the five domains and in the general factor for the bifactor model. Hierarchical (ω_h_) and total omegas (ω_t_) for each schema are also reported. The ratio ω_t_/ω_h_ expresses the variance component of the general factor in each observed variable in relation to the global variance due to all latent factors (Tommasi et al., 2015).

**Table 5:** Loadings on the first-order factors (λ_f_) and on the general factor (λ_g_) and corresponding significance (*p*-values).

**Young-L3 domains**	**Young-L3 schemas**	**λ_f_**	**λ_f_*p*-value**	**λ_g_**	**λ_g_*p*-value**	**ω_h_**	**ω_t_**	**ω_h_/ω_t_**
**NON-CLINICAL (*N* = 918)**								
Disconnection/Rejection	Emotional deprivation	0.49	<0.01	0.38	<0.01	0.15	0.38	0.38
	Abandonment	0.54	<0.01	0.55	<0.01	0.30	0.59	0.51
	Mistrust/Abused	0.75	<0.01	0.36	0.04	0.13	0.69	0.18
	Social isolation	0.46	<0.01	0.63	<0.01	0.40	0.60	0.66
	Defectiveness	0.37	0.03	0.78	<0.01	0.61	0.75	0.81
Impaired autonomy/Performance	Failure to achieve	**0.20**	**0.33**	0.80	<0.01	0.63	0.67	0.94
	Dependence	0.38	0.03	0.79	<0.01	0.62	0.76	0.81
	Vulnerability	0.61	<0.01	0.50	<0.01	0.25	0.63	0.40
	Enmeshment	0.55	<0.01	0.36	<0.01	0.13	0.43	0.30
Impaired autonomy/Performance	Subjugation	0.50	<0.01	0.70	<0.01	0.49	0.73	0.66
	Self-sacrifice	0.59	<0.01	**0.21**	**0.13**	0.04	0.39	0.12
	Approval-seeking	0.55	<0.01	0.51	<0.01	0.26	0.56	0.45
Impaired limits	Insufficient self-control	0.55	<0.01	0.60	<0.01	0.36	0.66	0.55
	Entitlement	0.90	<0.01	**0.20**	**0.21**	0.04	0.85	0.05
Overvigilance/Inhibition	Unrelenting standards	0.77	<0.01	**0.03**	**0.87**	<0.01	0.60	<0.01
	Emotional inhibition	0.58	<0.01	0.50	<0.01	0.25	0.59	0.42
	Negativism	0.58	<0.01	0.56	<0.01	0.32	0.65	0.48
	Self-punitiveness	0.67	<0.01	**0.29**	**0.08**	0.08	0.53	0.16
**CLINICAL (*N* = 148)**								
Disconnection/Rejection	Emotional deprivation	0.47	0.01	0.43	0.04	0.18	0.40	0.45
	Abandonment	**0.54**	**0.24**	0.70	0.04	0.48	0.77	0.63
	Mistrust/Abused	0.70	<0.01	**0.59**	**0.08**	0.34	0.83	0.41
	Social Isolation	**0.46**	**0.05**	0.69	<0.01	0.48	0.69	0.70
	Defectiveness	**0.33**	**0.32**	0.81	<0.01	0.65	0.76	0.86
Impaired autonomy/Performance	Failure to achieve	**0.12**	**0.82**	0.82	<0.01	0.67	0.68	0.98
	Dependence	**0.06**	**0.94**	0.86	<0.01	0.74	0.74	0.99
	Vulnerability	**0.42**	**0.42**	0.64	0.03	0.41	0.59	0.70
	Enmeshment	**0.50**	**0.32**	0.60	0.06	0.36	0.61	0.59
Impaired autonomy/Performance	Subjugation	**0.48**	**0.21**	0.72	<0.01	0.52	0.75	0.69
	Self-sacrifice	0.68	0.01	**0.32**	**0.48**	0.10	0.57	0.18
	Approval-seeking	**0.47**	**0.30**	0.64	0.04	0.40	0.63	0.65
Impaired Limits	Insufficient self-control	**0.54**	**0.09**	0.72	<0.01	0.51	0.80	0.64
	Entitlement	0.68	0.01	**0.58**	**0.06**	0.33	0.80	0.42
Overvigilance/Inhibition	Unrelenting standards	0.65	<0.01	**0.25**	**0.49**	0.06	0.48	0.13
	Emotional inhibition	0.50	0.11	0.67	<0.01	0.45	0.70	0.65
	Negativism	**0.50**	**0.25**	0.72	0.01	0.52	0.76	0.68
	Self-punitiveness	0.67	0.01	**0.52**	**0.13**	0.27	0.71	0.37

*Not significant loadings (p > 0.05) are reported on bold types. Hierarchical omega (ω_h_), total omega (ω_t_) and ratio between hierarchical and total omega (ω_h_ /ω_t_) are reported*.

The distributions of fit indices are affected by sample size and by the distribution of the measured characteristic in population (Yuan, 2005). Therefore, cutoffs of fit indexes cannot be considered as absolutely valid. In addition, the misfit of the models can be due to high covariance residuals instead of model misspecification. Covariance errors and model misspecification do not necessarily correspond (Hayduk et al., 2007). Therefore, not necessarily lower fit indexes indicate a misfit model. Factor loadings represent the quality of measurement of latent variables. Model with poor measurement quality (low factor loadings) can have a better fit than models with excellent measurement quality (high factor loadings). This phenomenon is called *reliability paradox* (Hancock and Mueller, 2011). On the basis of this paradox, McNeish and colleagues (McNeish et al., 2017) recommend to evaluate the validity of factor models not only on goodness of fit indexes, but also on the quality of their measures by reporting also factor loadings, because there is not a perfect correspondence between quality of measurement and fit indexes.

**Figure 1:**
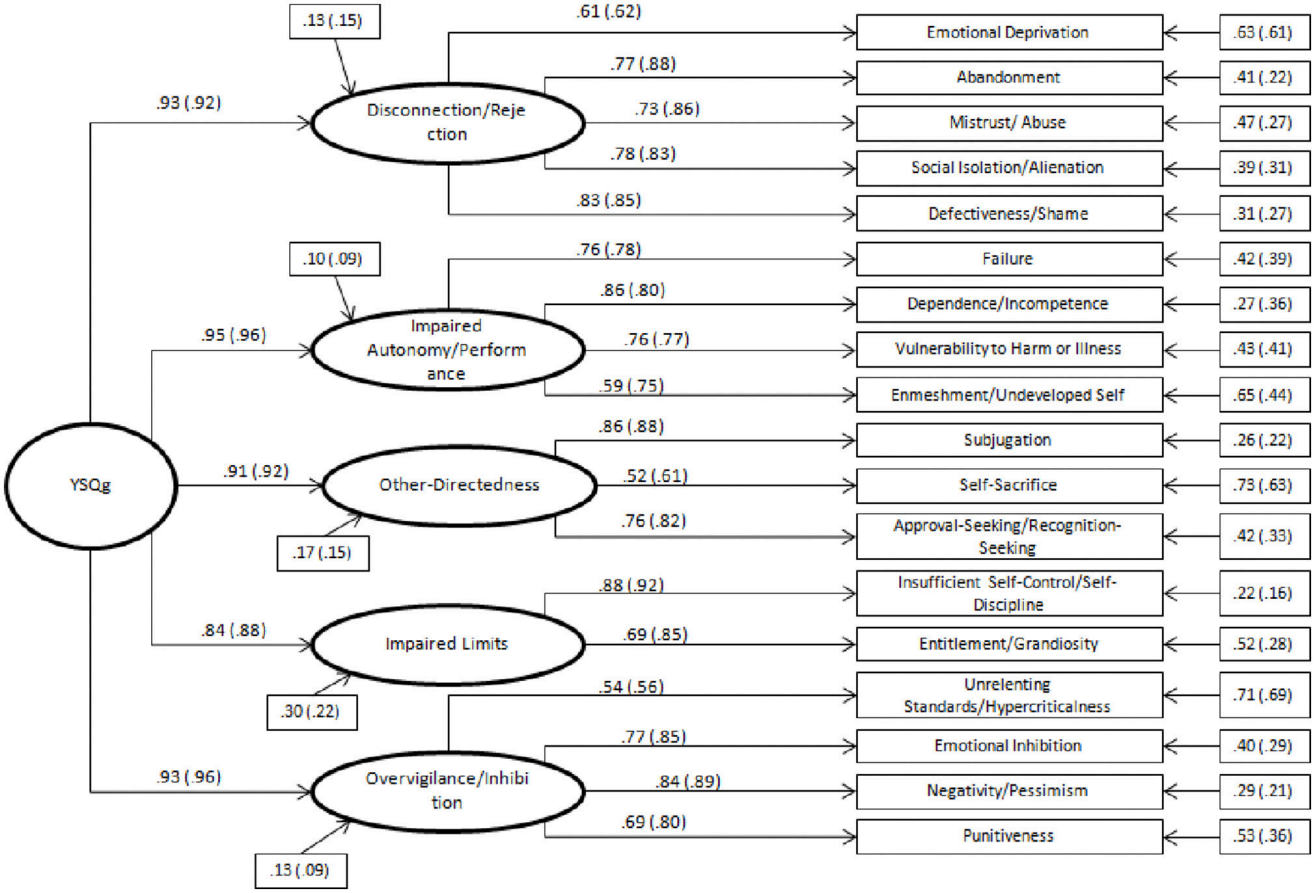
Path diagram of the second-order model of the YSQ-L3 (18 schemas and 5 domains) with reported standardized coefficients of first- and second-order loadings and residuals (clinical sample values are reported in parentheses). Residuals are reported in rectangles. All values are significant for *p* < 0.01.

In the second-order model, instead, all loadings of the five domains on schemas are significant both for the non-clinical and for the clinical groups. Figure 1 shows the path-diagram of the second-order model of the YSQ-L3.

### Measurement invariance of the YSQ-L3 between non-clinical and clinical groups

Table 6 shows the MG-CFA performed on the second-order model of the YSQ-L3. Because the second-order model has at work order loading, there is a version of the M2 model where the first-order loadings are fixed between groups (M2^*^) and a version where the first-order and the second-order loadings are fixed (M2^**^). All ΔCFI are lower than |0.01|, therefore the scalar invariance between the non-clinical and the clinical groups of the YSQ-L3 is confirmed. The difference between model M4 and M3 is however significant (Δ χ^2^ = 45.824, *df* = 5, *p* < 0.001). The means of the five domains of the YSQ-L3 are therefore significantly different between the non-clinical and the clinical group. All of the means of the five domains are higher in the clinical than in the non-clinical group.

**Table 6:** MG-CFA for testing measurement invariance of the YSQ-L3 between the non-clinical (*n* = 918) and the clinical (*n* = 148) sample.

**Models for measurement invariance**	**χ^2^**	**df**	**χ^2^/df**	**RMSEA**	**90% RMSEA**	**SRMR**	**TLI**	**CFI**	**ΔCFI**
M1	1906.673	26.000	7.333	0.109	0.104–0.114	0.066	0.800	0.830	
^M2*^	1911.471	273.000	7.002	0.106	0.102–0.111	0.068	0.810	0.831	**0.001**
^M2**^	1922.533	277.000	6.941	0.106	0.101–0.110	0.069	0.812	0.830	−**0.001**
M3	200.130	29.000	6.897	0.105	0.101–0.110	0.071	0.813	0.823	−**0.007**
M4	2045.954	295.000	6.935	0.106	0.101–0.110	0.089	0.812	0.819	

n.b.: M1,model for configural invariance;

M2* model for metric invariance (fixed first-order loadings);

M2** *, model for metric invariance (fixed first- and second-order loadings); M3 model for scalar invariance; M4, M3 with fixed means of YSQ-L3 domains for each group. ΔCFIs lower than |0.01| are in bold type*.

We therefore calculated the FPR for each schema and for each domain. On these calculations we estimated the percentage of risk in making FPs, multiplying the FPR ratio by 100, for both of the scores attained at the level of YSQ-L3 schemas and on factor scores of the five YSQ-L3 domains. Before estimating the FP risk for each YSQ-L3 schema, we transformed the raw scores of each schema in standardized scores. We estimated different distribution of standardized scores for the non-clinical and the clinical group. The cutoff values for the 0.05 and the 0.025 probability of FPs in the non-clinical group (first-type error) were used to estimate the probability values of TPs in the clinical group. We calculated the factor scores of the five domains to calculate the FPR for each domain. We estimated different distributions of standardized scores for the non-clinical and the clinical groups. The cutoff values for the 0.05 and the 0.025 probability of FPs in the non-clinical group (first-type error) were used to estimate the probability values of TPs in the clinical group. Table 7 shows the FP risk values for each YSQ-L3 schema and for each YSQ-L3 domain. The average FP risk value is 40.6 and 45.0% for the YSQ-L3 schemas, for the 5 and the 2.5% first-type error, respectively, while the average FP risk value for the YSQ-L3 domains is 24.2 and 18.2%, for the 0.05 and the 0.025 first-type error, respectively. FP risk is therefore lower when the factor scores for the five YSQ-L3 domains are used to discriminate between non-clinical and clinical subjects. According to Colquhoun (2014), the usual cutoffs for significance testing (0.05, 0.01 or 0.001) are somewhat misleading, because based on the assumption that if there are no significant differences between clinical and non-clinical subjects (null effect), therefore there is only a 5, 1 or 0.1% probability to judge an individual as a clinical subject while he is perfectly normal. However, this approach does not consider the power of the test or, in other words, the capacity of the psychological test to discriminate between clinical and non-clinical subjects. The test power is the probability to correct recognize the presence of disease in non-clinical subjects (true positives). If test power is not estimated, the correct identification of FPs is underestimated. Therefore, Colquhoun (2014) suggests to use the FPR instead of the usual null hypothesis significance test to determine its capacity to discriminate clinical from non-clinical subjects.

**Table 7:** False Positive Rate (FPR) risk values (in percentage values) for each YSQ-L3 schema and domain.

	**First-type errors**	
	**0.05**	**0.025**
**YSQ-L3 SCHEMAS**		
Emotional deprivation	33.1	38.2
Abandonment	30.3	31.6
Mistrust/ Abuse	38.1	42.5
Social isolation/Alienation	38.1	42.5
Defectiveness/Shame	33.1	34.6
Failure	36.3	48.1
Dependence/Incompetence	40.2	34.6
Vulnerability to harm or illness	45.1	31.6
Enmeshment/Undeveloped self	36.3	42.5
Subjugation	40.2	42.5
Self-sacrifice	55.2	55.2
Approval-seeking/recognition-seeking	30.3	48.1
Insufficient self-control/self-discipline	33.1	31.6
Entitlement/Grandiosity	51.4	38.2
Unrelenting standards/Hypercriticalness	51.4	78.7
Emotional inhibition	48.0	48.1
Negativity/Pessimism	38.1	42.5
Punitiveness	51.4	78.7
Average FPR	40.6	45.0
**YSQ-L3 DOMAINS**		
Disconnection/Rejection	21.6	16.3
Impaired autonomy/Performance	28.1	20.9
Other-directedness	29.1	25.2
Impaired limits	24.4	17.0
Overvigilance/Inhibition	17.9	11.7
Average FPR	24.2	18.2

## Discussion and conclusion

The YSQ-L3 (Young and Brown, 1994) is a self-report instrument, developed after a psychometric refinement of the previous version aimed at assessing the 18 EMS according to the ST theoretical framework. Its latent factor structure has not been consistently replicated (for a review, see Oei and Baranoff, 2007). In fact, almost all of the studies on the YSQ psychometric structure scrutinized the previous form (YSQ-L2) or the short form (YSQ-S3) and not the actual long form (YSQ-L3).

Knowledge of its factor structure could be useful both for researchers and for clinicians during assessment and treatment. The current study investigated the factor structure of the Italian YSQ-L3, its reliability, convergent validity with state/trait anxiety and depression measures, and measurement invariance across a large community and clinical groups.

CFAs analyses were conducted separately for the community and for the clinical groups, testing five different models: a single-factor model, a five correlated first-order factor model, a five uncorrelated first-order factor model, a bi-factor model and, finally, a second-order model, with the five first-order factors, according to Young's model, and a general second-order factor. Although the bi-factor model showed the best fit, both in the clinical group and the community group, some loadings of the five domains did not appear to be significant for their corresponding schemas, as posited by the original factor structure model, thus suggesting an inadequate fit. In the second-order model, instead, all loadings of the five domains on their schemas seemed to be significant both for the community and for the clinical groups. The second-order model was therefore preferred as it showed more adequate measurement properties than the bi-factor model for both of the groups. The original model proposed by Young et al. (2003) was therefore not confirmed in the current study.

Measurement invariance of the YSQ-L3 between community and clinical groups was subsequently tested for the second-order model. Results suggested that all ΔCFI were lower than |0.01|, thus supporting the scalar invariance between the community and the clinical groups. Since models M4 and M3 resulted significantly different, the means of the five domains of the YSQ-L3 appeared significantly different across the community and the clinical groups. All of the means of the five domains were higher in the clinical group than in the community group. The YSQ-L3 therefore appeared to be able to discriminate between the community and the clinical groups.

False positive risks indeed appeared lower when the factor scores of the five YSQ-L3 domains were used to discriminate between community and clinical individuals than when all of the 18 EMS were used. This result supported the ST model (Young et al., 2003), which posited that domains constructs are associated with psychopathology.

These results supply proof of the YSQ-L3 discriminant power and, consequently, of its validity. The average to high correlations between both the TDI and the STICSA supply additional proof of the YSQ-L3 capacity to measure psychopathology.

The ICC reliability estimates were in general insufficient or moderate and this could represent a problem for the YSQL-3.

This study bears various strengths. Firstly, it is one of the rare studies available about the YSQ-L3. YSQ-L3 is the most important version of the Young Schema Questionnaire and the most useful one as far as giving psychotherapists indications about patients' schemas. Secondly, at the best of our knowledge, this study is the most comprehensive one available as far as the validity of the Italian version of the YSQ-L3 is concerned. Third, participants were both community and clinical subjects.

An additional strength is supplied by the specific analyses that it reports for the first time, for example concerning he FPR risk values for each YSQ-L3 schema and domain.

Some limitations of the study should be highlighted. Firstly, the study uses a clinical group with different psychiatric diagnoses. An additional potential bias is that the clinical group included also individuals with comorbid personality disorders and individuals without them. Future research should thus investigate measurement invariance of the YSQ-L3 across different types of psychiatric disorders, such as clinical groups with only personality disorders and groups with only anxiety or depressive disorders. Examining whether the YSQ-L3 can discriminate between individuals with different personality disorders, eating disorders (Innamorati et al., 2015) or clusters of personality disorders could also be interesting.

Another limitation of this study concerns the lack of measures of other constructs related to EMS in the analysis of convergent validity, such as personality traits, attachment styles or functional/dysfunctional personal values (i.e., Balsamo et al., 2013; Picconi et al., 2018). Future studies should also investigate the responsiveness of the questionnaire in participants with psychiatric disorders after CBT or ST.

A further limitation concerns the numerous missing data. We tried to solve this problem in the best possible way. Anyway, particularly for this reason, a replication of the present study is welcomed.

In conclusion, the current study expanded previous knowledge beyond the inconclusive evidence about factor structure of the YSQ-L3, indicating a second-order model for the Italian version, and showing that it can be a valid and reliable instrument of measure than can be used in clinical practice and research.

## Ethics statement

In accordance with the Declaration of Helsinki, all participants provided written informed consent. Concerning ethics approval, the data collection process does not harm participants neither physically nor mentally.

## Author contributions

AS designed the study, assisted with data analyses, wrote part of the paper, and edited the final manuscript. MB assisted with the design of the study and data analyses, and wrote the most part of the paper. LC contributed in the analysis of the data and wrote part of the paper. VC recruited part of the sample. MS collaborated in editing the final manuscript and recruited part of the sample. GdF recruited part of the sample. DD recruited part of the sample and contributed in the analysis of the data. NM recruited part of the sample. IP recruited part of the sample. SP recruited part of the sample. MT assisted with the data analyses and collaborated in writing the manuscript.

### Conflict of interest statement

The authors declare that the research was conducted in the absence of any commercial or financial relationships that could be construed as a potential conflict of interest.

## References

[B1] American Psychiatric Association (2000). DSM-IV-TR: Diagnostic and Statistical Manual of Mental Disorders, Text Revision. Washington, DC: American Psychiatric Association.

[B2] American Psychiatric Association (2013). Diagnostic and Statistical Manual of Mental Disorders (DSM-5). Arlington, VA: American Psychiatric Publishing.

[B3] Bach B. Lee C. Mortensen E. L. Simonsen E. (2015). How do DSM-5 personality traits align with schema therapy constructs? J. Pers. Disord. 30, 502–529. 10.1521/pedi_2015_29_212 26305392

[B4] Balsamo M. (2010). Anger and depression: evidence of a possible mediating role for rumination. Psychol. Rep. 106, 3–12. 10.2466/PR0.106.1.3-12 20402420

[B5] Balsamo M. (2013). Personality and depression: evidence of a possible mediating role for anger trait in the relationship between cooperativeness and depression. Compr. Psychiatry 54, 46–52. 10.1016/j.comppsych.2012.05.007 22770718

[B6] Balsamo M. Carlucci L. Murdock K. K. Sergi M. R. Saggino A. (2015c). The mediating role of early maladaptive schemas in the relation between co-rumination and depression in youths. PLoS ONE 10:e0140177 10.1371/journal.pone.0140177 26488748 PMC4619064

[B7] Balsamo M. Carlucci L. Sergi M. R. Romanelli R. D'Ambrosio I. Fairfield B. (2016). A new measure for trait and state anxiety: the State Trait Inventory of Cognitive and Somatic Anxiety (STICSA). Standardization in an Italian population. Psicoterapia Cogn. Comportamentale 22, 229–232.

[B8] Balsamo M. Giampaglia G. Saggino A. (2014). Building a new Rasch-based self-report inventory of depression. Neuropsychiatr. Dis. Treat. 10, 153–165. 10.2147/NDT.S53425 24511231 PMC3913547

[B9] Balsamo M. Imperatori C. Sergi M. R. Belvederi Murri M. Continisio M. Tamburello A.. (2013b). Cognitive vulnerabilities and depression in young adults: an ROC curves analysis. Depress. Res. Treat. 2013:407602. 10.1155/2013/407602 24058734 PMC3766551

[B10] Balsamo M. Innamorati M. Van Dam N. T. Carlucci L. Saggino A. (2015a). Measuring anxiety in the elderly: psychometric properties of the state trait inventory of cognitive and somatic anxiety (STICSA) in an elderly Italian sample. Int. Psychogeriatr. 27, 999–1008. 10.1017/S1041610214002634 25592436

[B11] Balsamo M. Lauriola M. Saggino A. (2013). Work values and college major choice. Learn. Individ. Diff. 24, 110–116. 10.1016/j.lindif.2012.12.022

[B12] Balsamo M. Macchia A. Carlucci L. Picconi L. Tommasi M. Gilbert P.. (2015b). Measurement of external shame: an inside view. J. Pers. Assess. 97, 81–89. 10.1080/00223891.2014.947650 25157581

[B13] Balsamo M. Romanelli R. Innamorati M. Ciccarese G. Carlucci L. Saggino A. (2013a). The state-trait anxiety inventory: shadows and lights on its construct validity. J. Psychopathol. Behav. Assess. 35, 475–486. 10.1007/s10862-013-9354-5

[B14] Balsamo M. Saggino A. (2007). Test per l'assessment della depressione nel contesto italiano: un'analisi critica [Tests for depression assessment in Italian context: a critical review]. Psicoterapia Cogn. Comp. 13, 167–199.

[B15] Balsamo M. Saggino A. (2013). Il Teate Depression Inventory- Manuale [Teate Depression Inventory, Manual]. Florence: Hoegrefe.

[B16] Balsamo M. Saggino A. (2014). Determining a diagnostic cut-off on the teate depression inventory. Neuropsychiatr. Dis. Treat. 10, 987–995. 10.2147/NDT.S55706 24940062 PMC4051735

[B17] Bamelis L. L. Evers S. M. Spinhoven P. Arntz A. (2014). Results of a multicenter randomized controlled trial of the clinical effectiveness of schema therapy for personality disorders. Am. J. Psychiatry 171, 305–322. 10.1176/appi.ajp.2013.12040518 24322378

[B18] Caci H. Baylé F. J. Dossios C. Robert P. Boyer P. (2003). The Spielberger trait anxiety inventory measures more than anxiety. Eur. Psychiatry 18, 394–400. 10.1016/j.eurpsy.2003.05.003 14680715

[B19] Chen S. F. Wang S. Chen C. Y. (2012). A simulation study using EFA and CFA programs based the impact of missing data on test dimensionality. Expert Syst. Appl. 39, 4026–4031. 10.1016/j.eswa.2011.09.085

[B20] Cheung G. W. Rensvold R. B. (2002). Evaluating goodness-of-fit indexes for testing measurement invariance. Struct. Eq. Model. 9, 233–255. 10.1207/S15328007SEM0902_5

[B21] Colquhoun D. (2014). An investigation of the false discovery rate and the misinterpretation of p-values. R. Soc. Open Sci. 1:140216. 10.1098/rsos.140216 26064558 PMC4448847

[B22] Farrell J. M. Shaw I. A. Webber M. A. (2009). A schema-focused approach to group psychotherapy for outpatients with borderline personality disorder: a randomized controlled trial. J. Behav. Ther. Exp. Psychiatry 40, 317–328. 10.1016/j.jbtep.2009.01.002 19176222

[B23] Giesen-Bloo J. Van Dyck R. Spinhoven P. Van Tilburg W. Dirksen C. Van Asselt T.. (2006). Outpatient psychotherapy for borderline personality disorder: randomized trial of schema-focused therapy vs. transference-focused psychotherapy. Arch. Gen. Psychiatry 63, 649–658. 10.1001/archpsyc.63.6.649 16754838

[B24] Grös D. F. Antony M. M. Simms L. J. McCabe R. E. (2007). Psychometric properties of the State-Trait Inventory for Cognitive and Somatic Anxiety (STICSA): comparison to the State-Trait Anxiety Inventory (STAI). Psychol. Assess. 19, 369–381. 10.1037/1040-3590.19.4.369 18085930

[B25] Gude T. Hoffart A. (2008). Change in interpersonal problems after cognitive agoraphobia and schema-focused therapy versus psychodynamic treatment as usual of inpatients with agoraphobia and Cluster C personality disorders. Scand. J. Psychol. 49, 195–199. 10.1111/j.1467-9450.2008.00629.x 18352990

[B26] Halvorsen M. Wang C. E. Richter J. Myrland I. Pedersen S. K. Eisemann M.. (2009). Early maladaptive schemas, temperament and character traits in clinically depressed and previously depressed subjects. Clin. Psychol. Psychother. 16, 394–407. 10.1002/cpp.618 19479673

[B27] Hancock G. R. Mueller R. O. (2011). The reliability paradox in assessing structural relations within covariance structure models. Educ. Psychol. Meas. 71, 306–324. 10.1177/0013164410384856

[B28] Hawke L. D. Provencher M. D. Arntz A. (2011). Early maladaptive schemas in the risk for bipolar spectrum disorders. J. Affect. Disord. 133, 428–436. 10.1016/j.jad.2011.04.040 21621272

[B29] Hayduk L. Cummings G. Boadu K. Pazderka-Robinson H. Boulianne S. (2007). Testing! testing! one, two, three–testing the theory in structural equation models!. Pers. Individ. Dif. 42, 841–850. 10.1016/j.paid.2006.10.001

[B30] Hoffart A. Sexton H. Hedley L. M. Wang C. E. Holthe H. Haugum J. A. (2005). The structure of maladaptive schemas: a confirmatory factor analysis and a psychometric evaluation of factor-derived scales. Cogn. Ther. Res. 29, 627–644. 10.1007/s10608-005-9630-0

[B31] Hopkins W. G. (2000). Measures of reliability in sports medicine and science. Sports Med. 30, 1–15. 10.2165/00007256-200030010-00001 10907753

[B32] Hu L. T. Bentler P. M. (1999). Cutoff criteria for fit indexes in covariance structure analysis: conventional criteria versus new alternatives. Struct. Equation Model. 6, 1–55. 10.1080/10705519909540118

[B33] Innamorati M. Imperatori C. Meule A. Lamis D. A. Contardi A. Balsamo M.. (2015). Psychometric properties of the Italian Food Cravings Questionnaire-Trait-reduced (FCQ-T-r). Eating and Weight Dis. 20, 129–135. 10.1007/s40519-014-0143-2. 25069838

[B34] Innamorati M. Tamburello S. Contardi A. Imperatori C. Tamburello A. Saggino A.. (2013). Psychometric properties of the attitudes toward self-revised in Italian young adults. Depress. Res. Treatment 2013:209216. 10.1155/2013/209216 23878732 PMC3710639

[B35] Kriston L. Schäfer J. von Wolff A. Härter M. Hölzel L. P. (2012). The latent factor structure of Young's early maladaptive schemas: are schemas organized into domains? J. Clin. Psychol. 68, 684–698. 10.1002/jclp.21846 22528821

[B36] Lee C. W. Taylor G. Dunn J. (1999). Factor structure of the schema questionnaire in a large clinical sample. Cogn. Ther. Res. 23, 441–451.

[B37] Malogiannis I. A. Arntz A. Spyropoulou A. Tsartsara E. Aggeli A. Karveli S.. (2014). Schema therapy for patients with chronic depression: a single case series study. J. Behav. Ther. Exp. Psychiatry 45, 319–329. 10.1016/j.jbtep.2014.02.003 24650608

[B38] Mash E. J. Dozois D. J. A. (2003). Child psychopathology: a developmental-systems perspective, in Child Psychopathology, 2nd Edn. eds Mash E. J. Barkley R. A. (New York, NY: The Guilford Press), 3–74.

[B39] McGraw K. O. Wong S. P. (1996). Forming inferences about some intraclass correlation coefficients. Psychol. Methods 1, 30–46.

[B40] McNeish D. An J. Hancock G. R. (2017). The thorny relation between measurement quality and fit index cutoffs in latent variable models. J. Pers. Assess. 1–10. 10.1080/00223891.2017.1281286 28631976

[B41] Meins E. (2017). The predictive power of attachment. Psychologist 30, 21–24.

[B42] Meredith W. (1993). Measurement invariance, factor analysis and factorial invariance. Psychometrika 58, 525–543. 10.1007/BF02294825

[B43] Muris P. (2006). Maladaptive schemas in nonclinical adolescents: relations to perceived parental rearing behaviours, big five personality factors and psychopathological symptoms. Clin. Psychol. Psychother. 13, 405–413. 10.1002/cpp.506

[B44] Muthén L. K. Muthén B. O. (2012). Mplus Statistical Modeling Software: Release 7.0. Los Angeles, CA: Muthén & Muthén.

[B45] Nadort M. Arntz A. Smit J. H. Giesen-Bloo J. Eikelenboom M. Spinhoven P.. (2009). Implementation of outpatient schema therapy for borderline personality disorder with versus without crisis support of the therapist outside office hours: a randomized trial. Behav. Res. Ther. 47, 961–973. 10.1016/j.brat.2009.07.013 19698939

[B46] Oei T. P. S. Baranoff J. (2007). Young schema questionnaire: review of psychometric and measurement issues. Aust. J. Psychol. 59, 78–86. 10.1080/00049530601148397

[B47] Picconi L. Jackson C. J. Balsamo M. Tommasi M. Saggino A. (2018). Factor structure and measurement invariance across groups of the Italian Eysenck Personality Questionnaire-Short form (EPP-S). Pers. Individ. Dif. 123, 76–80. 10.1016/j.paid.2017.11.013

[B48] Rasch G. (1960). Probabilistic Models for Some Intelligence and Achievement Tests. Copenhagen: Danish Institute for Educational Research.

[B49] Ree M. J. French D. MacLeod C. Locke V. (2008). Distinguishing cognitive and somatic dimensions of state and trait anxiety: development and validation of the State-Trait Inventory for Cognitive and Somatic Anxiety (STICSA). Behav. Cogn. Psychother. 36, 313–332. 10.1017/S1352465808004232

[B50] Renner F. Arntz A. Leeuw I. Huibers M. (2013). Treatment for chronic depression using schema therapy. Clin. Psychol. Sci. Pract. 20, 166–180. 10.1111/cpsp.12032

[B51] Renner F. Lobbestael J. Peeters F. Arntz A. Huibers M. (2012). Early maladaptive schemas in depressed patients: stability and relation with depressive symptoms over the course of treatment. J. Affect. Disord. 136, 581–590. 10.1016/j.jad.2011.10.027 22119093

[B52] Riso L. P. du Toit P. L. Stein D. J. Young J. E. (2017). Cognitive Schemas and Core Beliefs in Psychological Problems: A Scientist-Practitioner Guide. Washington, DC: American Psychological Association.

[B53] Roberts K. E. Hart T. A. Eastwood J. D. (2016). Factor structure and validity of the State-Trait Inventory for Cognitive and Somatic Anxiety. Psychol. Assess. 28, 134–146. 10.1037/pas0000155 26011481

[B54] Saggino A. Balsamo M. Carlucci L. Sergi M. R. Da Fermo G. Déttore D. (2017). Analysis of the factor structure of the Italian version of the Young Schema Questionnaire L-3 in an Italian clinical and nonclinical sample: preliminary results of a multicenter study. G. Ital. Psicol. 44, 445–466. 10.1421/87349

[B55] Saggino A. Carlucci L. Sergi M. R. D'Ambrosio I. Fairfield B. Cera N. (2017). A validation study of the psychometric properties of the other as shamer scale−2. SAGE 7, 1–10. 10.1177/2158244017704241

[B56] Schermelleh-Engel K. Moosbrugger H. Müller H. (2003) Evaluating the fit of structural equation models: tests of significance descriptive goodness-of-fit measures. Methods Psychol. Res. Online 8, 23–74.

[B57] Schmidt N. B. Joiner T. E. Young J. E. Telch M. J. (1995). The Schema questionnaire: investigation of psychometric properties and the hierarchical structure of maladaptive schemas. Cognit. Ther. Res. 19, 295–321. 10.1007/BF02230402

[B58] Sempertegui G. A. Karreman A. Arntz A. Bekker M. H. (2013). Schema therapy for borderline personality disorder: a comprehensive review of its empirical foundations, effectiveness and implementation possibilities. Clin. Psychol. Rev. 33, 426–447. 10.1016/j.cpr.2012.11.006 23422036

[B59] Shrout P. E. Fleiss J. L. (1979). Intraclass correlations: uses in assessing rater reliability. Psychol. Bull. 86, 420–428. 18839484 10.1037//0033-2909.86.2.420

[B60] Stopa L. Thorne P. Waters A. Preston J. (2001). Are the short and long forms of the Young Schema Questionnaire comparable and how well does each version predict psychopathology scores? J. Cogn. Psychother. 15, 253–272.

[B61] Tommasi M. Pezzuti L. Colom R. Abad F. J. Saggino A. Orsini A. (2015). Increased educational level is related with higher IQ scores but lower g-variance: evidence from the standardization of the WAIS-R for Italy. Intelligence 50, 68–74. 10.1016/j.intell.2015.02.005

[B62] Van Dam N. T. Gros D. F. Earleywine M. Antony M. M. (2013). Establishing a trait anxiety threshold that signals likelihood of anxiety disorders. Anxiety Stress Coping 26, 70–86. 10.1080/10615806.2011.631525 22091946

[B63] Van Vlierberghe L. Braet C. Bosmans G. Rosseel Y. Bögels S. (2010). Maladaptive schemas and psychopathology in adolescence: on the utility of Young's schema theory in youth. Cogn. Ther. Res. 34, 316–332. 10.1007/s10608-009-9283-5

[B64] Vincent W. J. (1999). Statistics in Kinesiology. Champaign, IL: Human Kinetics.

[B65] Waller G. Kennerley H. Ohanian V. (2007). Schema-focused cognitive-behavioral therapy for eating disorders, in Cognitive Schemas and Core Beliefs in Psychological Problems. A Scientist-Practitioner Guide, eds Riso L. P. du Toit P. L. Stein D. J. Young J. E. (Washington, DC: American Psychological Association), 139–175.

[B66] West S. G. Finch J. F. Curran P. J. (1995). Structural Equation Models with Non normal Variables: Problems and Remedies. Structural Equation Modeling: Concepts, Issues, and Applications. Thousand Oaks, CA: Sage Publications.

[B67] Wright M. O. (2007). The long-term impact of emotional abuse in childhood: Identifying mediating and moderating processes. J. Emotional Abuse 7, 1–8. 10.1300/J135v07n02_01

[B68] Young J. E. (1994). Cognitive Therapy for Personality Disorders: A Schema-Focused Approach, Rev. Sarasota, FL: Professional Resource Press/Professional Resource Exchange.

[B69] Young J. E. Brown G. (1994). Young Schema-Questionnaire, in Cognitive Therapy for Personality Disorders: A Schema-Focused Approach, 2nd Edn., ed Young J. E. (Sarasota, FL: Professional Resource Press), 63–76.

[B70] Young J. E. Klosko J. S. Weishaar M. E. (2003). Schema Therapy. A Practitioner's Guide. New York, NY: The Guilford Press.

[B71] Yuan K. H. (2005). Fit indices versus test statistics. Multivariate Behav. Res. 40, 115–148. 10.1207/s15327906mbr4001_5 26822275

